# Cytokines and Soluble HLA-G Levels in the Acute and Recovery Phases of Arbovirus-Infected Brazilian Patients Exhibiting Neurological Complications

**DOI:** 10.3389/fimmu.2021.582935

**Published:** 2021-03-12

**Authors:** Renata Santos Almeida, Maria Lúcia Brito Ferreira, Paulin Sonon, Marli Tenório Cordeiro, Ibrahim Sadissou, George Tadeu Nunes Diniz, Maria de Fátima Pessoa Militão-Albuquerque, Rafael Freitas De Oliveira Franca, Eduardo Antonio Donadi, Norma Lucena-Silva

**Affiliations:** ^1^ Laboratory of Immunogenetics, Department of Immunology, Aggeu Magalhães Institute, Oswaldo Cruz Foundation, Recife, Brazil; ^2^ Hospital da Restauração Gov. Paulo Guerra, Recife, Brazil; ^3^ Ribeirão Preto Medical School, University of São Paulo, Ribeirão Preto, Brazil; ^4^ Department of Virology and Experimental Therapy, Aggeu Magalhães Institute, Oswaldo Cruz Foundation, Recife, Brazil; ^5^ Department of Collective Health, Aggeu Magalhães Institute, Oswaldo Cruz Foundation, Recife, Brazil

**Keywords:** Arbovirus, Zika (ZIKV), Chikungunya (CHIKV), Dengue (DENV), Neurological Complications, Cytokines, Chemokines

## Abstract

Severe neurological complications following arbovirus infections have been a major concern in seasonal outbreaks, as reported in the Northeast region of Brazil, where the same mosquito transmitted Zika (ZIKV), Dengue (DENV), and Chikungunya (CHIKV) viruses. In this study, we evaluated the levels of 36 soluble markers, including cytokines, chemokines, growth factors, and soluble HLA-G (Luminex and ELISA) in: i) serum and cerebrospinal fluid (CSF), during the acute phase and two years after the infection (recovery phase, only serum), ii) the relationship among all soluble molecules in serum and CSF, and iii) serum of infected patients without neurological complications, during the acute infection. Ten markers (sHLA-G, IL-10, IL-22, IL-8, MIP-1α, MIP-1β, MCP-1, HGF, VEGF, and IL-1RA) exhibited differential levels between the acute and recovery phases, with pronounced increases in MIP-1α (*P*<0.0001), MCP-1 (*P*<0.0001), HGF (*P*= 0.0001), and VEGF (*P*<0.0001) in the acute phase. Fourteen molecules (IL-1β, IL-2, IL-3, IL-4, IL-5, IL-6, IL-7, IL-9, IL-13, IL-15, IL-17A, IFN-α, TNF, and G-CSF) exhibited distinct levels between arbovirus patients presenting or not neurological complications. IL-8, EGF, IL-6, and MCP-1 levels were increased in CSF, while RANTES and Eotaxin levels were higher in serum. Soluble serum (IL-22, RANTES, Eotaxin) and CSF (IL-8, EGF, IL-3) mediators may discriminate putative risks for neurological complications following arbovirus infections. Neurological complications were associated with the presence of a predominant inflammatory profile, whereas in non-complicated patients an anti-inflammatory profile may predominate. Mediators associated with neuroregeneration (EGF and IL-3) may be induced in response to neurological damage. Broad spectrum immune checkpoint molecules (sHLA-G) interact with cytokines, chemokines, and growth factors. The identification of soluble markers may be useful to monitor neurological complications and may aid in the development of novel therapies against neuroinflammation.

## Introduction

In the 2015 outbreak, Brazil registered a significant increase in the number of hospitalizations and reported cases of microcephaly in babies, and Guillain Barré Syndrome (GBS), encephalitis and other neurological diseases in adults and children, with subsequent confirmation of their association with the *Flaviviridae* Zika virus (ZIKV) (1–7). ZIKV and the endemic genetically-related dengue virus (DENV) cause mild to severe flu-like symptoms; however, neurological manifestations are not the major complications of dengue infection (8–10). An overlap of cases with articular manifestations was also observed in the same epidemics, attributed to a new arbovirus (Chikungunya virus – CHIKV; *Togavirividae* family) (11, 12), which causes a broad spectrum of clinical manifestations, including temporary or chronic joint diseases and different types of neurological involvement (5, 13–15). During the outbreak, a single mosquito (*Aedes aegypti*) was responsible for the transmission of the three arboviruses (ZIKV, DENV, and CHIKV) at different combinations, and the highest concentration of Brazilian cases exhibiting severe neurological disorders was observed in Recife, capital of Pernambuco State (16).

Arbovirus-associated neurological diseases may be caused by the direct action of viruses, inflammatory reaction in nervous tissue or systemic metabolic alterations (encephalopathies), induced or not by the exacerbated systemic immune response (17). Neurological manifestations are consequence of virus-host complex interactions, including the viral neuroinvasive, neurotropic and neurovirulent properties and host susceptibility and host immune response (17, 18). Flaviviruses entry in cells by endocytosis after the binding of the viral envelop glycoprotein to the carbohydrate target-cell receptors (DC-SIGN and others), and by phagocytosis of the antibody-virus complex (17–19). Viruses penetrate the nervous system by different mechanisms, including the passage of the free-virus or infected immune cells through the hematogenous pathway across the blood-brain-barrier (BBB), direct infection of brain endothelial cells or transport of virus captured by peripheral sensory neural fibers by retrograde axonal pathway (20, 21). In addition to ZIKV, DENV and CHIKV may replicate in the nervous system, causing damage to the nervous tissue (22, 23). Only a fraction of infected patients develops neurological manifestations, exhibiting incubation time that vary according to the arbovirus (16).

The major inflammatory mediators associated with the acute phase of ZIKV patients include: i) increased levels of cytokines of the Th1 (IL-2), Th2 (IL-4, IL-13), Th17 (IL-17), and Th9 (IL-9) profiles (24), and ii) increased levels of the CXCL10/IP-10 chemokine (25). In addition, the infection of healthy peripheral blood mononuclear cells with ZIKV induces the production of soluble IL-6, IL-8 and the IL-9 cytokines, accompanied by a partial or complete lack of type I, II, and III interferons, essential for viral clearance and effective immune response (26). On the other hand, the infection of healthy monocytes with CHIKV infection increased soluble levels of IFN-α, IP-10, and IL-1RA (27). However, the role of virus-induced inflammatory mediators in BBB disruption and virus transport to the nervous system has not been fully understood.

Cytokines interact with several regulatory mediators of the immune system cells, including the immune checkpoint HLA-G molecule, which may reciprocally interact with cytokines (28, 29). As a well-recognized inhibitory protein, soluble HLA-G (sHLA-G) acts on NK, T, and B lymphocytes, monocytes, and dendritic cells ***via*** leukocyte receptor binding, such as ILT2, CD8, ILT4, and KIR2DL4 (30–32). An *in vitro* study with the human neural cell lines SK-N-SH/RA (neuroblastoma) and U373MG (astrocytoma) reported that herpes simplex virus 1 (HSV-1) and rabies virus (RABV) induce HLA-G expression in infected and in neighboring uninfected cells (33). Moreover, astrocytoma cells exhibited an upregulation of surface HLA-G1 (an isoform also released in fluids) after RABV infection, suggesting an infected cell mechanism to escape from the host immune response (33). After *in vitro* stimulation with cytomegalovirus (CMV) antigens, human peripheral blood leukocytes from infected patients increase sHLA-G levels compared to CMV negative cells (34). Little attention has been devoted to the role of HLA-G in acute emergent viral disorders, especially in biological samples (cerebrospinal fluid and serum) of patients during the infection and in the convalescent phase.

In this study, we expanded the investigation of the 2015 arbovirus (ZIKV, DENV, and CHIKV) outbreak that occurred in the Brazilian Northeastern State of Pernambuco, producing a myriad of neurological complications, to: i) perform a paired study evaluating the serum levels of 36 soluble molecules, including cytokines, chemokines, growth factors, cytokine receptors and HLA-G, at the acute phase and at the recovery phase of patients exhibiting neurological manifestations, ii) evaluate the profiles of these molecules in the cerebrospinal fluid (CSF) during the acute phase, iii) compare levels of soluble mediators between arbovirus infected patients exhibiting neurological manifestations with those who did not present neurological manifestations, and iv) study the reciprocal influence of these soluble molecules in serum and CSF, aiming to describe soluble mediators that may contribute in the pathogenesis of arbovirus-associated neurological disorders.

## Material and Methods

### Patients, Neurological Evaluation, and Sample Collection

We studied 185 patients referred to the Neurology Service at the Hospital da Restauração in Recife (Brazil) during 2015-2016 arbovirus epidemics, exhibiting suspicion of neurological complication of viral infections. At diagnosis, patients presented systemic features of infection, particularly fever, rash, myalgia, joint edema and arthralgia, and neurological symptoms of headache, altered consciousness, confusion, facial and limb weakness, paresis, abnormal reflexes, and sensory deficits, among others. All patients were submitted to neurological examination, electromyography, electroencephalography, and neuroimaging studies. Blood samples collected using Heparin Vacutainer tube (BD Biosciences, San Jose, CA) and CSF samples collected in a dry-sterile tube at the acute phase of the disease were sent under refrigeration to the arbovirus reference laboratory of the Brazilian Health Ministry at the Oswaldo Cruz Foundation in Pernambuco for viral diagnosis. Blood samples were centrifuged for 10 min at 1,500 x *g* for serum recovery as previously described (35). Serum and CSF aliquots were stored at ultralow temperature until testing for the presence of viral RNA and IgM antibodies against DENV, CHIKV, and ZIKV.

Among the 185 patients, 175 (median age=48 years, 95 women, 80 men) did present arbovirus-induced neurologic complications, which were classified by a senior neurologist (MLBF), according to their frequencies into: i) Guillain-Barre syndrome (GBS, 26%), ii) encephalitis spectrum disorders (ESD, 25%), which included encephalitis, meningoencephalitis, acute disseminated encephalomyelitis, rhombencephalitis, leukoencephalitis, and cerebellitis, iii) myelitis (14%), and iv) optic neuritis (11%), and several other syndromes that sum-up 24% cases. In 10 patients (median age = 61 years, seven women, three men), arbovirus neurological complications (e.g., disc hernia) were not confirmed. Eight of these patients were positive for DENV and CHIKV, one was positive only for DENV, and in the other patient, it was not possible to discriminate ZIKV from DENV. After two years of follow-up, additional peripheral blood samples were collected at the recovery phase (Major patients’ features are shown in **Table 1**). Detailed information about the demographic, laboratory, and clinical features of a larger group encompassing 210 patients (including the 175 of this series) was published by (16).

**Table 1:** Demographic and clinical characteristics of arbovirus patients with neurological complications.

Demographic and clinical data	Patients with neurological complications		Patients without neurological complications		
	(N/Total)	%		(N/Total)	%
**Age (years)**	**174/175**			**10/10**	
Median	48.00			61.00	
Mean ± SD	46.20 ± 18.45			63.50 ± 4.34	
**Gender**					
Male	80/175	45.71%		3/10	30.0%
Female	95/175	54.29%		7/10	70.0%
**Systemic manifestation**					
Fever	132/175	75.42%		8/10	80.0%
Rash	156/175	89.14%		3/10	30.0%
Myalgia	113/175	64.57%		8/10	80.0%
Arthralgia	131/175	74.80%		9/10	90.0%
**Neurological signs and symptoms**					
Headache	114/175	65.14%			
Altered consciousness	44/175	25.14%			
Limb weakness	123/175	70.28%			
Paraparesis	130/175	74.28%			
Quadriparesis	121/175	69.14%			
**Neurological manifestation**					
Encephalitis Spectrum Disorder	43/175	24.57%			
Guillain-Barré Syndrome	46/175	26.29%			
Myelitis	24/175	13.71%			
Optic Neuritis	19/175	10.86%			
Other Syndromes	43/175	24.57%			
**Virological Diagnosis**					
ZIKV^+^	27/174	15.52%		0/10	0.0%
DENV^+^	5/174	2.87%		1/10	10.0%
CHIKV^+^	45/174	25.86%		0/10	0.0%
CHIK^+^/ZIKV^+^	41/174	23.56%			
CHIK^+^/DENV^+^	5/174	2.87%		8/10	80.0%
ZIKV^+^/DENV^+^	3/174	1.72%			
CHIKV^+^/ZIKV^+^/DENV^+^	9/174	5.17%			
Negative/Inconclusive	39/174	22.41%		1/10	10.0%

ZIKV^+^: positive test for Zika virus; DENV^+^: positive test for Dengue virus; CHIKV^+^: positive test for Chikungunya virus.

### Study Design, Limitations, and Ethical Issues

To determine the profile of the soluble mediators at the acute (serum and CSF) and recovery phases (serum) of the arbovirus complications, we designed paired studies evaluating a total of 84 patients (median age = 46 years, 47 women, 37 men) infected by ZIKV, DENV, and CHIKV at several combinations. To evaluate the serum mediator profile during the acute phase, we compared these values with those observed at the convalescence phase, which would represent the patient’s steady state. To understand the possible role of serum mediators involved in the disruption of the BBB, we designed a paired study evaluating 12 (median age = 37 years, six women, six men) serum and CSF samples in the acute phase of diseases and studied the relationship among mediators between serum and CSF at the acute phase. Finally, to investigate the possible mediators associated with the development of neurological manifestations at the acute phase of the disease, we performed an unpaired study evaluating 136 serum samples (median age = 48 years, 75 women, 61 men) obtained from arbovirus infected patients with neurological complications and eight arbovirus-infected patients without neurological manifestations. To minimize sample size limitation, we performed correlation studies among mediators in each group (serum acute phase, serum recovery phase, CSF acute phase) separately, and discussed the differences between groups. In addition, we calculated ratios representing the proportion of mediators expressed in each group. The final conclusions were based on the ensemble of all these approaches.

This study was approved by the Ethics Committee of the Fiocruz of Pernambuco (CAAE: 63883517.4.0000.5190 and CAAE: 51106115.8.0000.5190), and all patients signed the informed consent form.

### Detection of Arbovirus Infection

Serum and CSF samples were tested for the presence of ZIKV, CHIKV, and DENV. RT-qPCR (Reverse transcription-quantitative polymerase chain reaction) was performed using reported methods for ZIKV (36) and for CHIKV (37) detection with minor modifications, and a well-established RT-qPCR protocol for DENV (38). In some patients, virus infection was also confirmed by virus isolation in Vero cells from serum and CSF samples, as previously published (35). Serological assays were performed for the detection of antibodies produced against each arbovirus, as reported in detail by (39). The assays were performed using DENV IgM-capture ELISA and DENV IgG indirect ELISA (Pan Bio, Inverness, Australia), and ELISA (IgM) and (IgG) for CHIKV (Euroimmun, Lübeck, Germany), following the manufacturer’s instructions. The ZIKV IgM-capture ELISA was performed with reagents provided by the Centers for Disease Control and Prevention (CDC; Fort Collins, CO), following the protocol described by (40). The DENV IgM and ZIKV-IgM tests were performed in parallel to evaluate cross-reactivity, and results were expressed by the ratio between the optical density (OD) at 450 nm of the test sample and the OD of the negative control. OD ratios below 2.0 were considered negative, ratios higher than 3.0 were positive, and ratios between 2.0 to 3.0 were inconclusive. Due to cross-reactivity among Flavivirus, the positivity for ZIKV-IgM was considered only when the OD for ZIKV was twice higher than the OD for DENV. Plaque reduction neutralization tests (PRNTs) were performed to investigate DENV and ZIKV cross-reactivity, as described by (39). It was considered a ZIKV primary infection when the patient tested positive for ZIKV-IgM and negative for DENV IgG by ELISA, or when the patient was positive exclusively for ZIKV in PRNT. Patients who tested positive for DENV IgG and ZIKV IgM by ELISA with PRNT positive for both ZIKV and DENV serotype were considered to have a ZIKV secondary infection.

### Soluble HLA-G Levels

sHLA-G (HLA-G1 and HLA-G5 isoforms) levels were measured using a sandwich ELISA, as previously described (41). The experiments were performed in duplicate for each sample and the results were expressed as ng/ml. ELISA results were obtained by using SpectraMax Plus 384 Microplate Reader (Molecular Devices, San Jose, CA), applying an absorbance filter of 450 nm.

### Cytokine, Chemokine, Growth Factor, and Cytokine Receptor Levels

We used the Human Cytokine Magnetic 35-Plex Panel (Invitrogen, Carlsbad, CA), following the manufacturer’s instructions, and data were acquired using MAGPIX^®^ Multiplexing Instrument (Luminex Corp., Austin, TX). We evaluated the following soluble mediators with their respective analytical sensitivity, according to the kit protocol: EGF (< 5 pg/ml), Eotaxin (< 0.5 pg/ml), FGF-basic (< 5 pg/ml), G-CSF (< 15 pg/ml), GM-CSF (< 0.5 pg/ml), HGF (< 5 pg/ml), IFN-α (< 5 pg/ml), IFN-γ (< 0.5 pg/ml), IL-1α (< 0.5 pg/ml), IL-1β (< 0.05 pg/ml), IL-1RA (< 0.5 pg/ml), IL-2 (< 0.5 pg/ml), IL-2R (< 25 pg/ml), IL-3 (< 5 pg/ml), IL-4 (< 1 pg/ml), IL-5 (< 0.5 pg/ml), IL-6 (< 0.5 pg/ml), IL-7 (< 5 pg/ml), IL-8 (< 0.5 pg/ml), IL-9 (< 0.5 pg/ml), IL-10 (< 0.5 pg/ml), IL-12 (< 1 pg/ml), IL-13 (< 5 pg/ml), IL-15 (< 10 pg/ml), IL-17A (< 1 pg/ml), IL-17F (< 10 pg/ml), IL-22 (< 10 pg/ml), IP-10 (< 0.5 pg/ml), MCP-1 (< 5 pg/ml), MIG (< 5 pg/ml), MIP-1α (< 5 pg/ml), MIP-1β (< 5 pg/ml), RANTES (< 5 pg/ml), TNF-α (< 0.5 pg/ml), and VEGF (< 0.05 pg/ml).

### Statistical Analysis

**Figure 1:**
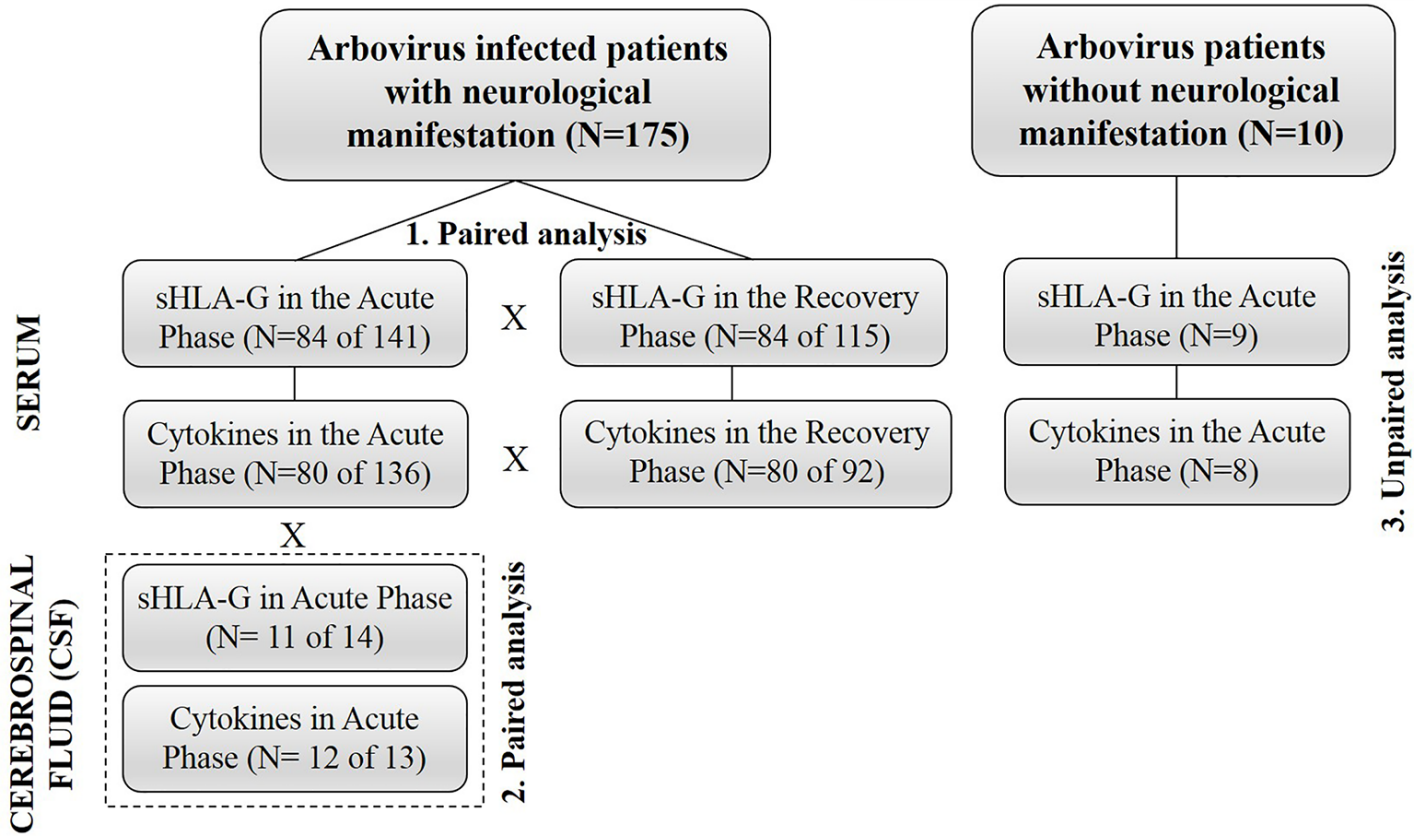
Flowchart summarizing major strategies used in the design of the study.

Demographic and clinical data were evaluated by a descriptive analysis. Considering that variables did not follow Gaussian distributions according to the Kolmogorov-Smirnov test, all analyses were performed using the non-parametric Mann-Whitney U and Wilcoxon Signed-Rank tests for independent and dependent variables, respectively. To assess correlations among variables, we used the Spearman’s correlation coefficient test. To evaluate the relative contribution of pairs of relevant mediators, we performed ratios between these mediators and compared these ratios in different situations, including: i) serum levels in acute versus recovery phases of patients exhibiting arbovirus infections with neurological complications; ii) serum levels in acute phase of arbovirus infection in patients presenting or nor neurological complications; iii) serum and CSF of patients in acute phase of arbovirus infection presenting neurological manifestations. *P*-value ≤ 0.05 was considered to be significant. Data were analyzed using SPSS V.20 (SAS Institute, Cary, NC) and GraphPad Prism V.5.01 (GraphPad Software, Inc.) softwares. A flowchart with all the experimental phases of the study is shown in **Figure 1**.

## Results

### Differences in Serum Molecule Levels Seen at the Acute and Recovery Phases in Patients With Arbovirus Infections Exhibiting Neurological Complications (Paired Study)

**Figure 2:**
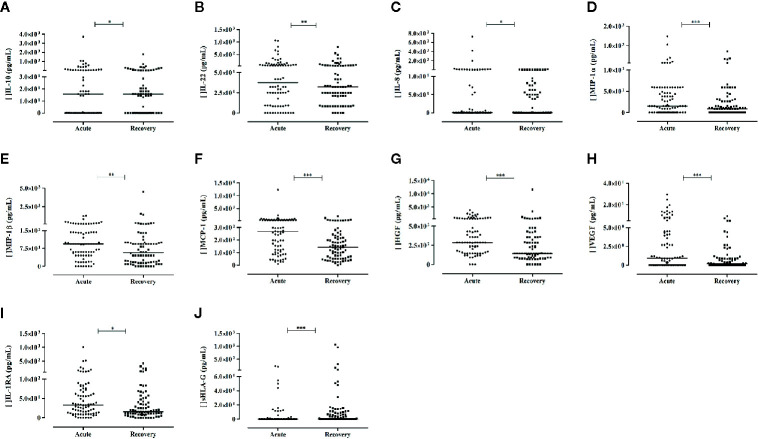
Soluble mediators exhibiting differential levels between acute phase and recovery phase of arbovirus patients presenting with neurological complications. Results from Wilcoxon Signed Rank Test. Cytokines: **(A, B)**; chemokines: **(C–F)**; growth factors: **(G, H)**; cytokine receptor: **(I)**; and sHLA-G: **(J)** P-values: *≤ 0.05; **< 0.01; ***< 0.0001. For **(A–I)**, N = 80; for **(J)**, N = 84.

The levels of nine mediators were increased in the acute phase when compared to the recovery phase, including the cytokines IL-10 and IL-22, the chemokines IL-8, MIP-1α, MIP-1β, and MCP-1, the growth factors HGF and VEGF, and the cytokine receptor IL-1RA. Notwithstanding, the most pronounced and significant differences were observed for MIP-1α (*P*< 0.0001), MCP-1 (*P*< 0.0001), HGF (*P*= 0.0001), and VEGF (*P*< 0.0001), representing, increases of 1.7-, 1.9-, 2- and 5-folds respectively, in the acute phase of the neurological manifestation (**Figures 2A–J**). Although the levels of IFN-α presented a 3-fold increase at the recovery phase in comparison to the acute phase, statistical significance was not reached.

The levels of sHLA-G were significantly lower in serum in the acute phase when compared to the recovery phase (*P*< 0.0001).

### Comparisons of Serum Versus Cerebrospinal Fluid Mediator Levels in the Acute Phase of Arbovirus Patients With Neurological Complications (Paired Study)

**Figure 3:**
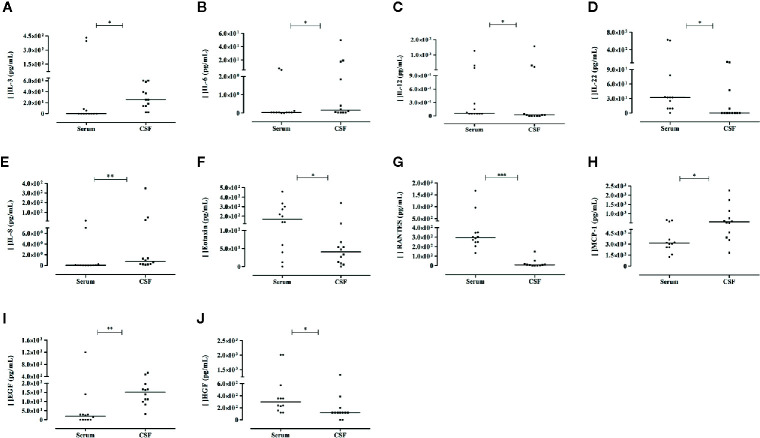
Differential levels of soluble mediators observed in serum and cerebrospinal fluid (CSF) of the same patient evaluated in the acute phase of arbovirus infection presenting neurological complications. Results from Mann-Whitney U Test (N = 12). Cytokine: **(A–D);** chemokines: **(E–H);** growth factors: **(I, J)**. *P*-values: *≤ 0.05; **< 0.01; ***< 0.0001.

Compared to serum levels, the CSF levels of IL-3, IL-8, EGF, IL-6, and MCP-1 were significantly higher (25.7-, 14.8-, 7.8-, 5.5-, and 1.8-folds, respectively), whereas the IL-22, RANTES, Eotaxin, HGF, and IL-12 levels were decreased in CSF. **Figures 3A–J** illustrates these results. We observed no correlations between serum and CSF levels, except for the positive correlation observed between IL-2 (rho = 0.921; *P*= 0.000) and GM-CSF (rho = 0.598; *P*= 0.040) levels. In CSF, sHLA-G levels were also decreased, and no significant difference was observed compared to serum levels in the paired samples.

### Correlations Among Soluble Mediator Levels in Serum and Cerebrospinal Fluid Observed in Arbovirus-Infected Patients Exhibiting Neurological Manifestations

Considering the known reciprocal influence of cytokines and sHLA-G (28, 29), and considering the possible reciprocal influence among all mediators analyzed in this study, we constructed several networks of interacting molecules presenting strong (rho ≥ 0.80) and significant *P*-values (<0.001). For sHLA-G, reciprocal correlations were performed using variables that presented *P*-values <0.01, due to its decreased expression in the acute phase.

Initially, to understand the relationship among mediators in patient steady state, we performed a set of correlations at disease recovery phase, excluding patients who exhibited comorbidities along the two-year follow-up. Then, we compared correlations among mediators in the acute and recovery phases.

**Figure 4:**
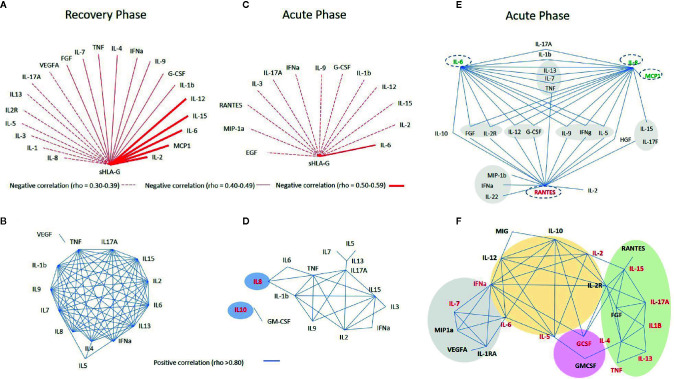
Networks constructed to evaluate the reciprocal influence among soluble mediators, evaluated in serum **(A–D)** and in cerebrospinal fluid (CSF) **(E)** of arbovirus infected patients exhibiting neurological manifestations during the acute phase of the infection **(C–E)** compared to the recovery phase **(A, B)**, and between patients exhibiting or not neurological manifestations in the acute phase of the infection **(F)**. In the networks shown in **(D, F)**, names in red indicate serum molecules which levels were significantly higher in acute phase compared to the recovery phase in patients exhibiting neurological manifestations. In the E network, names written in red mean decreased, whereas those in green denote increased levels in CSF when compared to serum levels. Red lines denote negative and blue lines positive correlations as evaluated by the Spearman correlation coefficient test.

At the recovery phase in serum, we observed: i) mild negative correlations between sHLA-G and IL-6 (rho = -0.520), MIP-1α (rho = -0.501), IL-2 (rho = -0.522), IL-12 (rho = -0.534), and IL-15 (rho = -0.515) and weak negative correlations (rho ranging from -0.30 to -0.49) between sHLA-G and the other 16 molecules (**Figure 4A**); ii) an intricate network of interactions among 12 cytokines (IL-1β, IL-2, IL-4, IL-6, IL-7, IL-8, IL-9, IL-13, IL-15, IL-17A, IFN-α, and TNF), exhibiting strong positive reciprocal correlations, and few other point interactions involving IL-5/IL-7, IL-5/IL-4, IL-5/IFN-α, and TNF/VEGF (**Figure 4B**).

At acute phase in serum: i) sHLA-G showed a weak negative correlations (rho between -0.30 and -0.39) with 12 molecules and a mild correlation only with IL-6 (rho = -0.448) (**Figure 4C**), and ii) a core of positive correlations was observed among TNF, IL-1β, IL-15, IL-17A, IL-2, and IL-9 cytokines (each cytokine forming nodes of 4–5 more interactions), and connected with this core IL-6, IL-8, IL-3, IFN-α, and IL-13 formed nodes of two to three interactions. The strongest positive correlation was observed between IL-10 and GM-CSF (rho = 0.933), and no other soluble molecules were directly correlated to these markers (**Figure 4D**).

At the acute phase in CSF, IL-8, IL-6, and RANTES formed the major cytokine network nodes, followed by chemokine and growth factor interactions, as well as specific correlations such as IL-6 with IL-10, and IL-8 with MCP-1, IL-15 and IL-17F (**Figure 4E**).

### Differences in Serum Molecule Levels in Patients With Arbovirus Infections Exhibiting Neurological Complications Compared to Those Without Neurological Manifestations (Unpaired Study)

**Figure 5:**
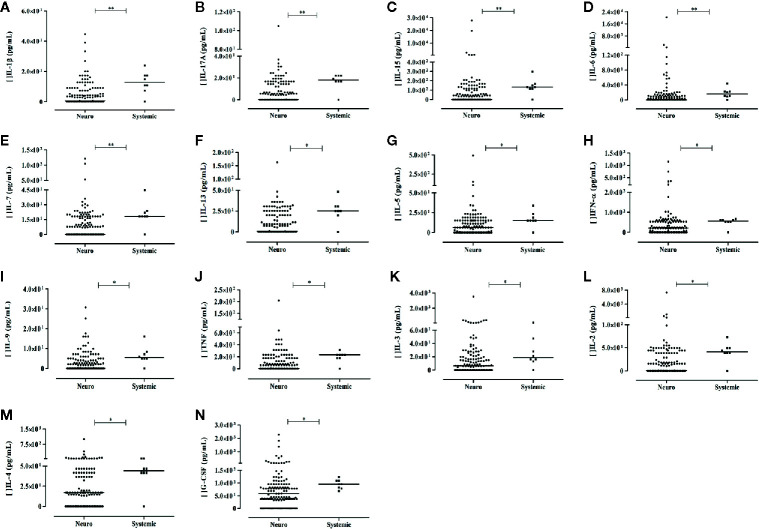
Serum soluble mediators exhibiting differential levels between arbovirus-infected patients exhibiting (Neuro) or not (Systemic) neurological complication. Results from Mann-Whitney U Test (Neuro – patients with neurological manifestation, N = 136; Control - patients without neurological manifestation, N = 8). Cytokines: **(A–M)**; growth factors: **(N)** *P*-values: *≤ 0.05; **< 0.01.

Thirteen cytokines (IL-1β, IL-2, IL-3, IL-4, IL-5, IL-6, IL-7, IL-9, IL-13, IL-15, IL-17A, IFN-α, and TNF) and the growth factor G-CSF exhibited higher levels in arbovirus-infected patient with no neurological complication compared to patients presenting neurological manifestations. These mediators are associated with inflammation (IL-1β, IL-6, TNF), stimuli for lymphocyte proliferation and differentiation (IL-2, IL-3, IL-7), antiviral activity (IL-17A, IL-15, IFN-α), and Th2 response (IL-9, IL-5, IL-13, and IL-4). The results are shown in **Figures 5A–N**. Noteworthy, the nine molecules that differentiated the acute phase profile from the recovery phase profile did not differentiate patients with neurological manifestation from those without these manifestations. sHLA-G levels did not differentiate patients exhibiting or not neurological complications (*P*> 0.05).

### Correlations Among Soluble Mediator Levels Observed in Serum of Arbovirus-Infected Patients Without Neurological Manifestations at the Acute Phase of the Disease

The network of interacting molecules for patients without neurological manifestation (22 molecules) diverged in terms of number of molecules and format from the one observed for patients with neurological manifestations (15 molecules). The major pattern of cytokine interaction included: i) a large central core of interactions encompassing IL-12, IL-10, IFN-α, IL-2, IL-2R, IL-5, and IL-6; ii) a small side core encompassing MIP-1α, IL-1RA, VEGF, and IL-7, connected to the central core through IL-6 and IFN-α; iii) a large side core formed by IL-15, IL-17A, IL-1β, TNF, IL-13, IL-4, FGF, and RANTES, connected to the central core though IL-2R and IL-2; and iv) point reciprocal correlations involving G-CSF, GM-CSF, and MIG (**Figure 4F**).

### Proportions Between Different Cytokine, Chemokine, and Growth Factor Levels in Arbovirus-Infected Patients

To investigate the contribution of molecules exhibiting differential levels between: i) the acute and recovery phases of patients exhibiting neurological manifestations, ii) serum and CSF in the acute phase of patients with neurological manifestations, and iii) serum of arbovirus infected patients with neurological manifestation and those without neurological manifestations, we evaluated the relative proportion of these molecules in each situation. In addition, we used the same strategy to calculate proportions between: i) inflammatory and anti-inflammatory molecules (IL-6, IL-1β, and others/IL-10), ii) molecules and their receptors (IL-1β/IL-1RA), iii) molecules with diverse mechanisms of action in relation with molecules exhibiting well-recognized roles on anti-viral defense (chemokines/IFN-α), iv) molecules pertaining to the same cytokine polarization profile (IL-22/IL-17), and v) molecules presenting a similar mechanism of action (chemokine/chemokine, growth factor/growth factor). The **Supplementary Table S1** illustrates major results

The proportions between the levels of the chemokines MIP-1α, MIP-1β, MPC-1, IP-10, and IFN-α, and the proportions between IL-8 and IL10 levels, and IL-6 and IL-10 levels were significantly higher in serum of acute phase of patients exhibiting or not neurological manifestations, and these mediators did not discriminate patients at risk for developing neurological manifestations. On the other hand, the proportions between the highly expressed acute phase IL-22 and chemokines RANTES and Eotaxin were considered to be markers for assessing risk of neurological manifestation, since their serum levels in acute phase were higher in patients presenting neurological manifestation than those without neurological features. The proportion between RANTES and IL-6 (336.3 vs 21.7, *P*=0.021), RANTES, and IL-8 (147.4 vs 7.8, *P*=0.013), Eotaxin and IL-6 (16.0 vs 1.3, *P*=0.010), and Eotaxin and IL-8 (12.2 vs 0.6, *P*=0.015) serum levels were higher in patients exhibiting neurological manifestations. In contrast, the ratios between IL-22 and IL-6 or IL-8 were not appropriated to discriminate the risk for neurological disease.

Considering the role of IL-22 and Th17 response in arbovirus disease, we further evaluated the ratio between IL-22 and IL-17 levels in the acute phase of the disease. Our results revealed a 75-fold increased level of the serum IL-22/IL-17A ratio in acute phase compared to recovery phase (*P* = 0.033), and a 3-fold increased level (*P* = 0.004) in patient with neurological manifestations in acute phase compared with patient without neurological manifestation, whereas no effect was observed using the IL-22/IL-17F ratio (*P* = 0.692). The proportion between IL-22 and TNF serum levels was also able to discriminate neurological disease (9.2 vs 3.0, *P* = 0.025) from non-neurological disease during acute phase.

In CSF, the proportions among IL-22, RANTES and Eotaxin levels were significantly lower compared to the proportions observed in serum. In contrast, the IL-1β/IL-10, IL-6/IL-10, IL-8/IL10, EGF/IL-10, and EGF/IL-12 ratios were higher in CSF when compared to their corresponding serum levels.

## Discussion

We assessed 36 soluble molecules in serum and CSF of arbovirus infected patients exhibiting neurological manifestations during the acute and recovery phases of the disease as well as in serum of arbovirus infected patients without neurological manifestations only during the acute phase of the disease. Since many of these molecules may present convergent, synergistic or divergent, antagonistic, shared or even specific effects, we used several strategies to discuss the results: i) lumping together all mediators, ii) evaluating specific situations; i.e., acute versus recovery phases in patients presenting neurological manifestations, acute phases of neurological and non-neurological patients, serum versus CSF levels, cytokine polarization profiles, and molecule clustering according to major functional group, and iv) correlations among variables and proportions between pairs of molecules in serum, CSF or both.

Initially, we evaluated the role of the immune checkpoint HLA-G molecule on arbovirus infections and its relationship with cytokines, chemokines, and growth factors. We observed a reduction of sHLA-G levels together with high cytokine and chemokine levels in the acute phase of arbovirus disease compared to recovery phase that would resemble the patient’s immune steady-state. Also, in the recovery phase, sHLA-G presented moderate negative correlations with a great number of serum molecules, including cytokines, chemokines and growth factors, corroborating the role of HLA-G on the modulation of the immune/inflammatory response (29, 30). In contrast, sHLA-G exhibited weak negative correlations with fewer molecules in the acute phase of arbovirus infection, except for IL-6, with which a moderate negative correlation was observed, suggesting that the HLA-G dependent modulation of inflammatory response was not efficient at the acute phase. In addition, decreased sHLA-G levels in serum were not associated with the development of neurological complications, since patients without neurological manifestation did not show differences in serum sHLA-G levels compared to who present neurological manifestations. High sHLA-G levels were reported in serum of patients with brain stem encephalitis caused by enterovirus 71(EV71) infection (42) and in CSF of patients with autoimmune disorders such as multiple sclerosis (43), suggesting that HLA-G is involved on the regulation of deleterious effects generated by inflammation in the central nervous system (CNS). Since sHLA-G was undetected or detected at low levels in CSF, it is not clear whether or not this finding is due to the direct/indirect viral effect or to decreased HLA-G regulatory factors. The great number of negative correlations with known HLA-G inducers like TNF and IFNs (44) indicates that both hypotheses may be plausible.

One of the major findings in the acute phase of arbovirus infections was the great number of chemokines exhibiting high levels in the serum. The chemokines IL-8 (CXCL8), involved in neutrophil recruitment, MIP-1α (CCL3), and MIP-1β (CCL4) macrophage and NK cell migration, and MCP-1 (CCL2) in the recruitment of inflammatory monocytes to the site of infection (45), were elevated in serum in the acute phase regardless of the presence or not of neurological manifestations. Increased IL-8, MCP-1, MIP-1α, and MIP-1β serum levels have also been reported in the serum of ZIKV-infected patients compared to uninfected individuals (25). In the acute phase of disease, the MCP-1 and IP-10 levels were increased in the serum of pregnant women infected with ZIKV carrying fetuses with developmental abnormalities compared to those carrying healthy fetuses (46). In the present study, we observed that the ratios between the chemokines MCP-1, MIP-1α, and MIP-1β and the natural antiviral IFN-α were high in the serum at acute phase; however, they were not associated with risk of neurological involvement, since the serum proportions were similar to those observed in patient without neurological manifestations; nevertheless, the protective role of IFN-α levels for neurological manifestation is noted in the proportion IFN-α/MCP-1. In addition, the amount of serum IL-8 was 1.11-fold increase in the acute compared to the recovery phase, but 14.8-fold increase in CSF compared to serum levels, suggesting that IL-8 and neutrophil recruitment to the inflammatory site may have a role in the neuropathy caused by arbovirus infections (45). In a recent study, neutrophil extracellular traps were observed in mouse during acute CHIKV infection as an effective mechanism of infection control (47). Evidence also indicates that high levels of IL-8 are associated with cognitive impairment in HIV encephalitis (48, 49), and high levels of MCP-1 have been associated with the severity of neural damage in patients with Herpes simplex virus (HSV) encephalitis (50). In this context, we observed a strong positive correlation between IL-8 with the inflammatory cytokines IL-6, TNF, and IL-1β in the acute phase of the disease in patients with neurological manifestation. Further increase in the IL-6 and IL-1β serum levels were influenced by the low sHLA-G level. In contrast, in infected patients without neurological manifestation, none of these inflammatory molecules had a reciprocal correlation, and IL-8 had no or weak influence on the expression of these molecules. On the other hand, the IL-8 and IL-6 levels in CSF were high, and these mediators shared strong positive correlation with other 12 molecules as well as with each other. Finally, the high level of MCP-1 in CSF was exclusively and positively correlated with IL-8 levels, suggesting a cooperative action on the induction of the neurological damage.

Regarding growth factors, high levels of HGF and VEGF were observed in serum during the acute phase of the neurological and non-neurological diseases, differing from the levels reported for infants with congenital neurological disease (46), but agreeing with results reported for patients with ZIKV (VEGF and G-CSF) when compared to non-infected individuals (25). Nevertheless, the strong positive correlation between VEGF and IL-1RA serum levels was observed exclusively in non-neurological disease, suggesting a distinct mechanism of inflammation control in the neurological disorder. Elevated levels of serum VEGF were observed in children with viral encephalitis (51), in untreated patients with HIV-associated encephalopathy (52), and in patients with severe dengue (53, 54). On the hand, EGF levels were increased in CSF of patients with neurological manifestations and did not show significant correlations with other molecules. If, by one hand, VEGF and HGF increase vascular permeability contributing to inflammation and nerve damage, and permitting disruption of the blood-brain barrier (BBB) (52, 55, 56), on the other hand, EGF has a neuroprotective role in neurological diseases (57–59). Besides its immunotolerance effect, *in vitro* studies report that sHLA-G may down-regulate VEGF, EGF and HGF (44, 60), and the decreased levels of sHLA-G in serum and CSF may contribute to the increased HGF and VEGF serum levels and EGF CSF levels in patients with neurological manifestations. Therefore, the undesired VEGF and HGF effect on BBB may be counterbalanced by the neuroprotective effect of EGF in patients with neurological complications.

The ratio between IL-22 and IL-17 levels played a key role on the identification of arbovirus-infected patients prone to develop neurological manifestations, since: i) IL-22 levels were significantly increased in serum, but not in CSF, ii) IL-22 did not present a strong correlation with any other serum molecule in acute or recovery phases, iii) IL-22 was not included in the core CSF molecule correlation network, iv) IL17A serum levels were increased in patients without neurological manifestations when compared to those presenting neurological manifestations, v) the median IL-22/IL-17A ratio was 75-folds high in serum in acute phase compared to recovery phase, and vi) no similar ratio was observed using the IL-22/IL-17F in patients without neurological involvement. IL-22 is produced by several immune cells during inflammation, acting in tissue regeneration, cell survival and proliferation, and also in host defense in epithelial barrier by the recruitment of leukocyte facilitating its antimicrobial effect (61). In mice, high level of circulating IL-22 was observed in the fourth day of West Nile Virus (WNV) infection and was associated with leucocyte infiltration into CNS. *IL22^-/-^* mice had low viral load in CNS when WNV was introduced by subcutaneous route compared to high viral load when introduced by intracranial inoculation, confirming that IL-22 regulates the production of chemoattractants by microvascular endothelial cells in BBB (62). Both IL-22 and IL-17 cytokines produced by T_H_17 cells were reported to induce MCP-1 secretion by BBB-endothelial cells, increasing BBB permeability. IL-17 was reported to increase IL-6 and IL-8 production by BBB-endothelial cells (63), and it is also responsible for the high CSF levels of IL-6 and IL-8. Considering the BBB permeability is a crucial early event for neurological damage, the ratio of IL-22/IL-17A levels in serum seemed to be an accurate serological marker for neurological assessment risk in arbovirus infections.

Although variable levels of IL-10 have been reported in individuals infected by ZIKV along the acute phase of the infection (23), in our series IL-10 levels were elevated in both groups of patients in the acute phase of the infection. Similarly, increased IL-10 levels were observed in the serum of patients with other viral encephalitis (HSV), in which it was associated with lower disease severity (50). IL-10 regulates innate and Th1 and Th2 adaptive immune responses by inhibiting the macrophage phagocytic activity, T cell activation and synthesis of pro-inflammatory cytokines; therefore, depending on the time of the infection, increased IL-10 levels may impair the viral clearance, but concurrently can ameliorate the tissue damage (64). Besides the increased IL-10 levels in serum of patients with neurological manifestations, we observed that that the ratio between IL-6 and IL-10 and between IL-8 and IL-10 were high in serum and extremely high in CSF during the acute phase of the disease, suggesting that IL-10 levels did not efficiently down regulate the effects of IL-6 and IL-8 during the acute phase. Furthermore, we observed that serum IL-10 levels were exclusively correlated to GM-CSF levels during the acute phase in patients with neurological manifestations. Considering that: i) GM-CSF is also produced by Th17 cells, ii) IL-22 and IL-17 stimulate BBB-endothelial cells to produce the IL-8, MCP-1 chemokines and IL-6, iii) IL-6 also stimulates naïve T-cell to differentiate in Th22 cells, further increasing the levels of IL-22 in serum, the ensemble of these mediators may favor virus entry into CNS (65–67).

Noteworthy, none of the nine overexpressed molecules (MIP-1α, MIP-1β, MCP-1, IL-8, VEGF, HGF, IL-1RA, IL-10, and IL-22) in serum of patients with neurological manifestation during the acute phase showed differential expression in patients without neurological manifestations. Besides, patients without neurological manifestations also exhibited a distinct profile, regarding the overexpression of inflammatory (IL-1β, IL-6, TNF), cell proliferation (IL-2, IL-3, IL-7), antiviral (IL-17A, IL-15, IFN-α), Th2 response (IL-9, IL-5, IL-13, IL-4), and growth factor (G-CSF) molecules. The correlation analyses showed that neurological and non-neurological manifestations exhibited distinct immune regulation patterns, as it has been reported for viral or immune-mediated encephalitis and cerebral malaria (50, 68, 69). Among serum molecules identified in non-neurological and neurological diseases in the acute phase of the infection, 12 (IL-1β, IL-6, TNF, IL-2, IL-7, IL-17A, IL-15, IFN-α, IL-5, IL-13, IL10, and GM-CSF) were shared by both networks. Particular differences in these networks included the finding that IL-8 exclusively modulated several molecules in the network constructed for neurological disease and exhibited an exclusive positive correlation with IL-10 and GM-CSF. Taken together, these results corroborate associations with mediators related to neutrophil recruitment and those associated with changes in BBB permeability.

Considering molecule networks constructed for non-neurological manifestations, G-CSF was linked to IFN-α and IL-15, which were augmented in serum during the acute phase of the disease. G-CSF levels are increased in the serum of patients with ZIKV compared to uninfected Brazilian subjects (25). G-CSF is involved on the differentiation and proliferation of neutrophils and monocytes (70); and may play a role in T cell tolerance (71, 72). Since IFN-α and IL-15 are involved in the antiviral response (73), the high serum levels may be related to a more effective immune response against the virus in patients without neurological manifestations. Contrasting with the network observed for neurological manifestations (exclusive and important positive correlation between IL-10 and GM-CSF in serum), in patients without neurological features, we observed a strong positive correlation between IL-10 and several molecules exhibiting antiviral activity (IFN-α and G-CSF), anti-inflammatory effect (IL-12 and MIG), pleiotropic effect (IL-6), and with the Th2 immune response (IL-5, IL-2, and IL-2R). These findings suggest that the Th2 polarization profile has an immunoregulatory role on preventing neurological manifestations. In experimental models, Th2 cytokines (IL-2, IL-4, IL-5, and IL-10) protected JEV-infected mice against viral encephalitis, and high CSF IL-10 levels were associated with protection against brain damage (73). Overall, our results corroborate the hypothesis that the Th2 response is associated with protection against neurological manifestations in arbovirus infections, as evidenced by: i) the modulation of the immune response exerted by IL-10, ii) the correlation between IL-6, IL-2, and IL-5, but not IL-17A, and iii) the high levels of IL-4, IL-2, and IL-5 molecules in the serum of non-neurological patients.

The studies comparing CSF and serum levels in patients with neurological manifestations revealed interesting results, supporting the role of pro-inflammatory, anti-inflammatory, and neuroprotective factors. Evidence supporting the predominance of inflammatory rather than anti-inflammatory factors includes: i) increased levels of IL-6, IL-8, MCP-1, EGF, and IL-3 in CSF, while IL-22, IL-12, HGF, eotaxin, and RANTES were increased in serum, predominating inflammatory markers in CSF, ii) although IL-6 is involved in neuroinflammation and neurological damage, it is also considered to be a neurotrophin, exhibiting a role in neurogenesis and neuroregeneration (74). Despite its neuroprotective role, in this study IL-6 seems to exert a rather inflammatory effect, since the increased CSF level was associated with increased IL-8 and MCP-1 levels (45), iii) unbalanced IL-6/IL-10, IL-8/IL-10, IL-1β/IL-10, and IL-1β/IL-1RA ratios were observed in CSF ​​compared to the serum; also corroborating the predominant effect of inflammatory molecules in CSF. Unbalanced IL-1β/IL-10 and IL-1β/IL-1RA ratios associated with elevated levels of IL-10 and IL-1RA in CSF were also reported to be associated with a better prognosis of acute encephalitis caused by viruses or by other mechanisms (50), and iv) the elevated serum levels of RANTES and eotaxin indicate inflammation mediated by eosinophils and basophils, which may explain the skin symptoms presenting in some arbovirus infected patients (50, 75). In contrast, the high levels of EGF in CSF may be associated with a neuroprotective effect in arbovirus patients exhibiting neurologic manifestations. Indeed, EGF presents a neuroprotective role in neurological disorders (69–71). Besides EGF, IL-3 may also exert a protective role against brain damage in arbovirus infection, since IL-3 CSF levels were more than 25-folds higher than in serum, and few weak correlations were observed with other CSF molecules. IL-3 is a multi-colony stimulatory factor that may be produced by some neuronal cells in physiological conditions (76) and by CD4-infiltrating T cells in experimental autoimmune encephalitis (77). Since peripheral blood mononuclear cells expressing membrane-bound HLA-G, but no sHLA-G, may also induce a shift towards a Th2 profile inducing the expression of IL-3 (27), and since we just observed decreased sHLA-G in CSF, it is possible that the major source of IL-3 is from infiltrating cells in neurological manifestations.

Besides reporting evidence, supporting the idea of direct arbovirus damage to nervous tissues, the findings of the present series shed light to further understand the pathogenesis of neurological complications at central and peripheral nervous system after exposure to biological agents, expanding the current idea that the immune response against the biological agent is the major pathogenic event for the peripheral nervous diseases (e.g., *Campylobacter jejuni* and GBS (78, 79). Considering that we observed the presence of the arbovirus (RT-PCR for ZIKV, DENV, CHIKV) and the presence of IgM antibodies (against ZIKV, DENV, CHIKV) in the CSF in concomitance with the neurologic manifestations, both direct invasion and the inflammation triggered by the immunological response against the viruses may account for the arbovirus-mediated neuropathy.

Concluding, we reported the role of soluble mediators in serum (IL-22, RANTES, Eotaxin) and in CSF (IL-8, EGF, IL-3) exhibiting a potential role to discriminate putative risks for neurological complication development in patients infected by arboviruses. The appearance of neurological complications may be induced by the presence of a predominant inflammatory profile, whereas in non-complicated patients an anti-inflammatory profile may predominate. Indeed, increased levels of pro-inflammatory mediators may also operate in other viral infections accompanied by neurological manifestation (50, 68), as well as in immune-mediated neurological diseases (multiple sclerosis, neuromyelitis, GBS, encephalitis, others). Mediators associated with neuroregeneration like EGF and IL-3 may be induced in response to damage or to prevent further damage. Broad spectrum immune checkpoint molecules (HLA-G) interact with cytokines, chemokines, and growth factors; however, their roles on acute viral infections must be further scrutinized. The identification of soluble mediators can be further used to monitor/manage arbovirus complications as well as to develop novel therapies against neuroinflammation.

## Data Availability Statement

The original contributions presented in the study are included in the article/**Supplementary Material**. Further inquiries can be directed to the corresponding author.

## Ethics Statement

The studies involving human participants were reviewed and approved by the Ethics Committee of the Aggeu Magalhães Institute (Approved protocols: CAAE 63883517.4.0000.5190 and CAAE 51106115.8.0000.5190). The patients/participants provided their written informed consent to participate in this study.

## Author Contributions

ED and NL-S conceived, designed, and applied for grants to perform the study. MF, MC, MM-A, RF, and RA collected samples and provided clinical and laboratory information. RA, PS, and IS performed the laboratory experiments. RA processed the data and wrote the original manuscript draft. RA and GD performed the statistical analysis. RA, MF, MC, MM-A, RF, ED, and NL-S performed the data curation. All authors have critically read and edited the manuscript. All authors contributed to the article and approved the submitted version.

## Funding

This study was supported by the Conselho Nacional de Desenvolvimento Científico e Tecnológico (CNPq, grants #440760/2016-0 and #302060/2019-7); the Coordenação de Aperfeiçoamento de Pessoal de Nível Superior (CAPES, grants # 88881.130769/2016-01 and Finance Code 001), and the Aggeu Magalhães Institute/Oswaldo Cruz Foundation. RA was supported by CAPES (Grant# 88887.187948/2018-00), PS was supported by CAPES (Grant# 88887.124115/2014-00) and is currently sponsored by Oswaldo Cruz Foundation (Fiocruz, Grant #INOVA FIOCRUZ/02/2019 PDJ), and NL-S is a CNPq scholarship holder (Grant #310364/2015-9; #310892/2019-8).

## Conflict of Interest

The authors declare that the research was conducted in the absence of any commercial or financial relationships that could be construed as a potential conflict of interest.
