# Supplementary material for: Cytokines and Soluble HLA-G Levels in the Acute and Recovery Phases of Arbovirus-Infected Brazilian Patients Exhibiting Neurological Complications
Source: Front Immunol. 2021 Mar 12;12:582935. doi: 10.3389/fimmu.2021.582935 (PMC7994272; doi:10.3389/fimmu.2021.582935)
Supplement: Supplementary file 1 [file Table_1.docx]

Supplementary Material

Table S1 – Cytokine and chemokines ratios in different conditions of arbovirus patients presenting or not neurological manifestations.

|  | **Patients Conditions** | | | | | | | |  |
| --- | --- | --- | --- | --- | --- | --- | --- | --- | --- |
| **Ratio** | **Acute** | **Recovery** |  | **Case** | **Control** |  | **Serum** | **CSF** |  |
|  |  |  | ***P-*value^1^** |  |  | ***P-*value** |  |  | ***P-*value** |
| **IL-1β/IL-10** | N= 65^2^  0.168^3^ | N= 67  0.196 | 0.888 | N= 121  0.173 | N= 8  0.332 | 0.079 | N= 12  0.429 | N= 11  3.900 | **0.035** |
| **IL-1β/IL-1RA** | N= 75  0.0015 | N= 72  0.0025 | 0.216 | N= 130  0.026 | N= 8  0.258 | **0.014** | N= 12  0.001 | N= 12  0.003 | **0.016** |
| **IL-6/IL-10** | N= 55  0.302 | N= 67  0.209 | **0.013** | N= 121  0.240 | N= 8  0.310 | 0.315 | N= 12  0.604 | N= 11  2.900 | **0.012** |
| **IL-6/IL-4** | N= 55  0.302 | N= 50  0.256 | 0.490 | N= 108  0.318 | N= 8  0.439 | 0.650 | N= 8  2.417 | N= 10  2.417 | 0.252 |
| **IL-8/IL-10** | N= 65  0.446 | N= 67  0.295 | **0.044** | N= 121  0.519 | N= 8  0.680 | 0.414 | N= 12  0.968 | N= 11  20.900 | **0.003** |
| **IFN-α/MCP-1** | N= 80  0.000 | N= 79  0.000 | **0.052** | N= 136  0.032 | N= 8  0.132 | **0.015** | N= 12  0.000 | N= 12  0.000 | 0.542 |
| **IFN-α/MIP-1α** | N= 57  0.490 | N= 48  1.795 | **0.011** | N= 110  1.073 | N= 7  1.589 | 0.427 | N= 9  0.000 | N= 9  0.000 | 0.911 |
| **HGF/VEGF** | N= 51  166.300 | N= 44  243.200 | **0.017** | N= 104  179.300 | N= 8  157.700 | 0.477 | N= 10  256.200 | N= 12  111.500 | **0.023** |
| **IL-8/VEGF** | N= 51  0.060 | N= 44  13.800 | **0.036** | N= 104  11.080 | N= 8  11.110 | 0.501 | N= 10  0.060 | N= 12  0.307 | **0.051** |
| **IL-22/IL-17A** | N= 51  405.300 | N= 47  4.440 | **0.033** | N= 102  10.920 | N= 6  3.715 | **0.004** | N= 5  2,472 | N= 6  1.220 | **0.017** |
| **IL-22/IL-17F** | N= 66  0.195 | N= 70  0.195 | 0.692 | N= 119  0.220 | N= 8  0.275 | 0.545 | N= 9  0.164 | N= 6  0.111 | 0.314 |
| **IL-22/IL-13** | N= 40  4.135 | N= 39  3.425 | **0.042** | N= 88  4.139 | N= 7  2.775 | **0.031** | N= 3  3.503 | N= 5  1.107 | 0.143 |
| **IL-22/Eotaxin** | N= 78  0.756 | N= 79  0.605 | 0.716 | N= 134  0.910 | N= 8  3.299 | **0.026** | N= 11  0.162 | N= 11  0.000 | **0.064** |
| **IL-22/TNF** | N= 55  84.386 | N= 49  5.558 | 0.254 | N= 109  9.239 | N= 7  3.043 | **0.025** | N= 10  1476.000 | N= 11  0.000 | **0.002** |
| **IL-22/IL-8** | N= 73  131.443 | N= 69  96.182 | 0.470 | N= 129  4.802 | N= 8  2.076 | 0.321 | N= 12  455.324 | N= 12  0.000 | **0.000** |
| **IL-22/IL-6** | N= 57  317.276 | N= 48  7.708 | 0.061 | N= 111  11.200 | N= 8  5.713 | 0.076 | N= 11  1108.241 | N= 12  0.000 | **0.000** |
| **EGF/IFN-γ** | N= 64  5.522 | N= 68  4.699 | 0.380 | N= 118  7.117 | N= 8  11.977 | 0.706 | N= 9  6.820 | N= 12  4190 | **0.054** |
| **EGF/IL-10** | N= 65  0.849 | N= 67  0.565 | 0.206 | N= 121  0.894 | N= 8  2.151 | 0.837 | N= 12  0.995 | N= 11  11,173 | **0.003** |
| **EGF/IL-12** | N= 78  0.616 | N= 78  0.462 | 0.428 | N= 136  1.084 | N= 8  2.019 | 0.496 | N= 12  0.945 | N= 7  4469 | **0.036** |
| **EGF/GM-CSF** | N= 60  2.327 | N= 60  1.634 | 0.259 | N= 112  2.392 | N= 7  5.278 | 0.298 | N= 9  3.450 | N= 7  2793 | 0.089 |
| **RANTES/IL-10** | N= 65  24.211 | N= 67  13.772 | 0.818 | N= 121  18.157 | N= 8  6.214 | 0.458 | N= 12  7637.249 | N= 11  470.996 | **0.004^*^** |
| **RANTES/IL-1RA** | N= 74  7.237 | N= 72  13.192 | **0.038** | N= 130  8.254 | N= 8  5.836 | 0.578 | N= 12  7.923 | N= 12  0.197 | **0.001** |
| **RANTES/IFN-α** | N= 55  69.543 | N= 50  29.426 | 0.096 | N= 109  39.916 | N= 8  5.466 | 0.098 | N= 11  22137.333 | N= 12  578.805 | **0.001** |
| **Eotaxin/IL-10** | N= 65  1.174 | N= 67  1.178 | 0.978 | N= 121  1.319 | N= 8  0.433 | 0.274 | N= 12  4338.292 | N= 11  2338.931 | 0.487 |
| **Eotaxin/IL-1RA** | N= 74  1.986 | N= 72  2.079 | 0.505 | N= 130  2.072 | N= 8  0.555 | 0.097 | N= 12  3.126 | N= 12  1.177 | 0.160 |
| **Eotaxin/IFN-α** | N= 55  3.331 | N= 50  1.873 | 0.181 | N= 109  2.196 | N= 8  0.427 | **0.029** | N= 11  15184 | N= 12  5026 | 0.074 |
| **IP-10/IL-10** | N= 65  0.643 | N= 67  0.291 | 0.712 | N= 121  0.409 | N= 8  0.526 | 0.762 | N= 12  40.618 | N= 11  95.900 | 0.379 |
| **IP-10/IL-1RA** | N= 74  0.245 | N= 72  0.387 | **0.043** | N= 130  0.247 | N= 8  0.483 | 0.142 | N= 12  0.068 | N= 12  0.137 | 0.266 |
| **IP-10/IFN-α** | N= 55  2.051 | N= 50  0.243 | **0.019** | N= 109  0.643 | N= 8  0.435 | 0.623 | N= 11  174.167 | N= 12  159.833 | 1.000 |
| **MIP-1α/IL-10** | N= 65  0.608 | N= 67  0.565 | 0.078 | N= 121  0.608 | N= 8  0.767 | 0.799 | N= 12  263.694 | N= 11  4057.138 | 0.169 |
| **MIP-1α/IL-1RA** | N= 74  0.660 | N= 72  0.710 | 0.544 | N= 130  0.649 | N= 8  0.654 | 0.913 | N= 12  0.716 | N= 12  1.222 | 0.443 |
| **MIP-1α/IFN-α** | N= 55  0.993 | N= 50  0.524 | **0.013** | N= 109  0.681 | N= 8  0.611 | 0.466 | N= 11  1982.988 | N= 12  3268.708 | 0.413 |
| **MIP-1β/IL-10** | N= 65  5.353 | N= 67  1.124 | 0.152 | N= 121  2.446 | N= 8  2.116 | 0.914 | N= 12  2030.821 | N= 11  1996.400 | 0.379 |
| **MIP-1β/IL-1RA** | N= 74  2.089 | N= 72  1.770 | 0.572 | N= 130  2.040 | N= 8  2.118 | 0.891 | N= 12  1.819 | N= 12  1.127 | 0.347 |
| **MIP-1β/IFN-α** | N= 55  12.653 | N= 50  1.812 | **0.001** | N= 109  4.362 | N= 8  1.649 | 0.287 | N= 11  8299.663 | N= 12  3327.333 | 0.059 |
| **MCP-1/IL-10** | N= 65  14.401 | N= 67  5.011 | 0.068 | N= 121  13.766 | N= 8  9.731 | 0.507 | N= 12  6059.134 | N= 11  25960.207 | 0.118 |
| **MCP-1/IL-1RA** | N= 74  7.866 | N= 72  5.831 | 0.243 | N= 130  9.149 | N= 8  5.088 | 0.489 | N= 12  5.630 | N= 12  17.509 | 0.078 |
| **MCP-1/IFN-α** | N= 55  31.208 | N= 50  5.518 | **0.000** | N= 109  18.506 | N= 8  7.646 | 0.062 | N= 11  31963.667 | N= 12  67837.833 | 0.288 |
| **Eotaxin/IL-6** | N= 57  5210.983 | N= 48  13.298 | **0.048** | N= 111  16.025 | N= 8  1.250 | **0.010** | N= 11  4701 | N= 12  121.600 | **0.031** |
| **Eotaxin/IL-8** | N= 73  787.215 | N= 69  406.228 | 0.843 | N= 129  12.272 | N= 8  0.582 | **0.015** | N= 12  4150 | N= 12  53.660 | **0.038** |
| **Eotaxin/MCP-1** | N= 80  0.177 | N= 79  0.333 | **0.022** | N= 136  0.177 | N= 8  0.107 | 0.158 | N= 12  0.533 | N= 12  0.061 | **0.009** |
| **Eotaxin/EGF** | N= 48  1.484 | N= 46  1.635 | 0.591 | N= 101  1.468 | N= 7  0.198 | **0.002** | N= 7  2.803 | N= 12  0.195 | **0.001** |
| **RANTES/IL-6** | N= 57  2515.603 | N= 48  263.199 | **0.020** | N= 111  336.339 | N= 8  21.684 | **0.021** | N= 11  8532 | N= 12  32.930 | **0.0003** |
| **RANTES/IL-8** | N= 73  2160.786 | N= 69  1537.829 | 0.935 | N= 129  147.429 | N= 8  7.831 | **0.013** | N= 12  5150 | N= 12  12.620 | **< 0.0001** |
| **RANTES/MCP-1** | N= 80  0.940 | N= 79  2.085 | **0.001** | N= 136  0.950 | N= 8  1.082 | 0.780 | N= 12  1.012 | N= 12  0.018 | **0.0006** |
| **RANTES/EGF** | N= 48  7.334 | N= 46  12.258 | 0.226 | N= 101  7.977 | N= 7  3.238 | 0.146 | N= 7  8.920 | N= 12  0.040 | **0.008** |
| **IL-3/EGF** | N= 48  0.096 | N= 46  0.385 | **0.002** | N= 101  0.250 | N= 7  0.352 | 0.377 | N= 7  0.330 | N= 12  0.142 | 0.966 |
| **IL-3/IL-10** | N= 65  0.000 | N= 67  0.119 | 0.222 | N= 121  0.212 | N= 8  0.362 | 0.190 | N= 12  0.000 | N= 11  1411 | **0.002** |
| **IL-3/IL-6** | N= 57  0.000 | N= 48  1.605 | **0.006** | N= 111  1.211 | N= 8  1.084 | 0.576 | N= 11  0.000 | N= 12  60.840 | **0.014** |
| **IL-3/IL-8** | N= 73  0.000 | N= 69  0.480 | **0.008** | N= 129  0.151 | N= 8  0.493 | 0.267 | N= 12  0.000 | N= 12  28.060 | **0.008** |
| **IL-6/IL-17A** | N= 51  0.679 | N= 47  0.674 | 0.751 | N= 102  0.764 | N= 7  0.949 | 0.946 | N= 5  2.231 | N= 6  2.407 | 0.714 |

Note: ^1^*P*-value calculated from Mann-Whitney U test. Acute and Recovery – Serum of patients in acute and recovery phases of arbovirus infection with neurological impairment; Case and Control – Serum of patients in acute phase of arbovirus infection presenting (case) or not (control) neurological complications; Serum and CSF – Serum and cerebrospinal fluid (CSF) of patients in acute phase of arbovirus infection presenting neurological manifestations. ^2^N – number of patient’s samples; ^3^Median values of the ratio evaluated. **P*-value calculated from Student T-test, data with normal distribution.
